# Alginate/Poly(*γ*-glutamic Acid) Base Biocompatible Gel for Bone Tissue Engineering

**DOI:** 10.1155/2015/185841

**Published:** 2015-10-04

**Authors:** Wing P. Chan, Fu-Chen Kung, Yu-Lin Kuo, Ming-Chen Yang, Wen-Fu Thomas Lai

**Affiliations:** ^1^Department of Radiology, Wan Fang Hospital, Taipei Medical University, Taipei 110, Taiwan; ^2^Department of Radiology, School of Medicine, College of Medicine, Taipei Medical University, Taipei 110, Taiwan; ^3^Department of Health Healing and Health Marketing, Kainan University, Taoyuan 338, Taiwan; ^4^Department of Mechanical Engineering, National Taiwan University of Science and Technology, Taipei 106, Taiwan; ^5^Department of Materials Science and Engineering, National Taiwan University of Science and Technology, Taipei 106, Taiwan; ^6^Graduate Institute of Clinical Medicine and Graduate Institute of Medical Sciences and Graduate Institute of Biomaterials, Taipei Medical University, Taipei 110, Taiwan

## Abstract

A technique for synthesizing biocompatible hydrogels by cross-linking calcium-form poly(*γ*-glutamic acid), alginate sodium, and Pluronic F-127 was created, in which alginate can be cross-linked by Ca^2+^ from Ca–*γ*-PGA directly and *γ*-PGA molecules introduced into the alginate matrix to provide pH sensitivity and hemostasis. Mechanical properties, swelling behavior, and blood compatibility were investigated for each hydrogel compared with alginate and for *γ*-PGA hydrogel with the sodium form only. Adding F-127 improves mechanical properties efficiently and influences the temperature-sensitive swelling of the hydrogels but also has a minor effect on pH-sensitive swelling and promotes anticoagulation. MG-63 cells were used to test biocompatibility. Gelation occurred gradually through change in the elastic modulus as the release of calcium ions increased over time and caused ionic cross-linking, which promotes the elasticity of gel. In addition, the growth of MG-63 cells in the gel reflected nontoxicity. These results showed that this biocompatible scaffold has potential for application in bone materials.

## 1. Introduction

Tissue engineering aims to create wound-covering biomaterials for skin-related diseases and biological body parts as alternatives to transplanted harvested tissues and organs. One example, hydrogel, has been exploited for medical and pharmaceutical applications for scaffolding or covering for the last three decades [1]. The three-dimensional networks of hydrogels offer excellent chemical and mechanical stability [2]. The junction zones within hydrogels can be chemical or physical cross-links. Chemical hydrogels have permanent network structures formed by irreversible covalent bonds, but the covalently cross-linked networks may lead to toxic effects from free unreacted covalent cross-linking agents or organic solvents. Physical hydrogels are nonpermanent network structures formed by various reversible cross-links. These can be ionic bonds, hydrogen bonds, or hydrophobic interactions [3]. However, the reversible cross-links have rather poor mechanical properties for some applications. Therefore, enhancing their strength and durability could make physical hydrogels more applicable as biomaterials.

Alginate is used extensively as a hydrogel component because of its many advantages, such as hydrophilicity, high swellability, nontoxicity, and easy preparation [4, 5]. Alginate hydrogels also have the biocompatibility required for successful medical and pharmaceutical use; their high water content enhances the superhydrophilic diffusing surface, and low interfacial tension minimizes transport resistance for the adsorption or release of solutes [6]. Alginate is a natural substance that can be designed and developed in various medical and pharmaceutical ways. However, its gelation process has been inconvenient and not suitable for practical use.

Polyglutamic acid (PGA) is a natural linear polymer which is copolymerized from the amino acid glutamic acid, which is formed by peptide bonds between the amino group and the carboxyl group at the end of the glutamic acid side chain. It can be synthesized by bacilli such as* Bacillus subtilis* and obtained from bacterial fermentation [7].

Pluronic F-127 is a triblock copolymer of poly(ethylene oxide)-poly(propylene oxide)-poly(ethylene oxide) (PEO-PPO-PEO), which has a nominal molecular weight of 12 500 and an average formula of EO_99_PO_67_EO_99_ [8]. Aqueous solutions of F-127 at concentrations of 15–20 wt% and higher are liquid when refrigerated, but gel upon warming [9]. This gel formation process has been extensively studied [10–13]. It has been shown that gel formation occurs due to progressive dehydration of the polymer micelles with increase in temperature, leading to increased chain entanglement.

We aimed to study biocompatibility and cytocompatibility of different proportions of F-127 with alginate-calcium-PGA (described as A-CP-F) in hydrogels prepared via a casting method. There are two advantages to using this blend as a bone tissue engineering material.

The blended hydrogel can provide mechanical strength, so A-CP-F gels would be more suitable for clinical application than a simple hydrogel. In this study, these A-CP-F gels were subject to hydrophilic tests, tensile tests, protein adsorption, and blood coagulation tests to demonstrate the clinical applicability of the A-CP-F matrix.

## 2. Materials and Methods

### 2.1. Materials

Sodium alginate (Acros, USA) with molecular weight about 22 kD was used without further purification. Sodium-form and calcium-form *γ*-PGA (Vedan Enterprise Corporation, Taiwan) with molecular weight about 1 MD, calcium chloride (Shimakyu Chemical Co., Japan), MTT (3-(4,5-dimethylthiazol-2-yl)-2,5-diphenyltetrazolium bromide; 5 mg/mL in DMEM culture medium and serum), ascorbic acid 2-glucoside (AA2G) (Hayashibara Co. Ltd., Japan), human serum albumin (HAS) (Mw: 66000), and human plasma fibrinogen (HPF) (Mw: 341000) (Calbiochem, USA) were used. Pluronic F-127 (Sigma, USA) was used without further purification. All solutions of F-127 were made by using the cold method described by Hyun et al. [9], whereby F-127 was added to water at ambient temperature before dissolution at refrigeration temperature.

### 2.2. Preparation of Gel Samples

A 1.5 wt% homogeneous alginate solution was prepared by dissolving sodium alginate powder in deionized water at room temperature for 24 h under stirring. Calcium-PGA powder was dissolved in deionized water at room temperature under stirring for 2 h to form a homogeneous solution of 3 wt%. Then F-127 solution was mixed with Ca-PGA solution at seven ratios, and 20 mL of the alginate solution was cast onto a glass plate. Finally, 20 mL of the Ca-PGA-F-127 solution was poured over the alginate to form a hydrogel. For comparison, sodium alginate hydrogel was soaked in either 10 wt% CaCl_2_, 3 wt% Na-PGA, or 3 wt% Ca-PGA aqueous solution. The resulting hydrogels were labeled as in Table 1. All these hydrogels were rinsed with deionized water to remove residual solutions and dried at 60°C for 1 d in an oven.

### 2.3. Swelling Ratios Test

We measured the swelling behavior of the gels under different pH with normal saline solution and different temperatures of deionized water, and samples of 1 × 1 cm^2^ were dried in an oven for 2 h at 105°C [14]. Then, the samples were placed in a humidifying chamber at 37°C with 90% relative humidity and weighed at specific time points. The swelling ratios of the samples were calculated as follows:

(1) $$\begin{array}{c} \text{SR}=\frac{W_{\text{wet}}}{W_{\text{dry}}}, \end{array}$$

where *W*_wet_ and *W*_dry_ represent the weights of the film in the wet and dry states, respectively.

### 2.4. Water Retention Capacity Test

A piece of swollen hydrogel was weighed (*W*_*i*_) and placed in a tube. After centrifugation at 2000, 8000, and 16000 g at 25°C for 5 min, the sample was carefully removed and weighed (*W*_*t*_) [14]. The water retention percentage was calculated using the following formula:

(2) $$\begin{array}{c} \text{WR}\%=\frac{W_{t}-W_{d}}{W_{i}-W_{d}}\times 100, \end{array}$$

where *W*_*d*_ is the dry weight of the sample.

### 2.5. Tensile Tests

The tensile strength and breaking elongation of the blended hydrogels were measured with a tensile tester (MTS 810; Material Test System, USA) according to ASTM D882-02. The dry samples were prepared by vacuum drying at 40°C overnight. The specimens were cut into a specific dog-bone shape (11.5 cm long, 2.5 cm wide at the ends, and 0.6 cm wide in the middle). The thickness of each specimen was measured. The measurement was conducted at a crosshead speed of 10 mm/min under a tensile preload of 10 kg.

### 2.6. Adsorption of Proteins

The adsorption of HAS and HPF was measured. A piece of hydrogel of 1 × 1 cm^2^ was immersed in 5 mL of pH 7.4 PBS containing 2 mg/dL HSA or HPF at 37°C for 24 h under 100 rpm shaking. Afterwards, the samples were gently taken out and rinsed five times with PBS, followed by placing them in 1 wt% aqueous solution of sodium dodecyl sulfate (SDS), and shaken for 60 min at room temperature to remove the protein adsorbed on the surface. The protein content of each sample was measured using the BCA reagents (Pierce). The absorbance at 562 nm was measured using a spectrometer to calculate the concentration of protein [15].

### 2.7. Evaluation of Platelet Adhesion

The determination of platelet adhesion and thrombus formation followed published procedures [16]. Briefly, platelet-rich plasma (100 *μ*L) was placed on test hydrogels (1 × 1 cm^2^) at 37°C for 30 and 60 min, respectively. The platelet counts at baseline (about 4 × 10^5^ *μ*L^−1^) and after adhesion were measured using a hematology analyzer (CA-620; Medonic, Sweden). The extent of platelet adhesion relative to the platelet-rich plasma control was calculated as follows:

(3) $$\begin{array}{c} \text{Platelet adhesion }\left(\%\right)=\frac{n_{0}-n_{t}}{n_{0}}\times 100, \end{array}$$

where *n*_0_ is the count at baseline and *n*_*t*_ is the count after adhesion.

### 2.8. Blood Coagulation Test

The* in vitro* coagulation times, including the activated partial thrombin time (APTT), were measured using a blood coagulation tester (CA-50; Sysmex Corp., Japan). A sample of 1 × 1 cm^2^ was placed in 50 *μ*L platelet-poor plasma and incubated at 37°C for 1 min, followed by adding 50 *μ*L APTT reagent. After incubation for 3 min, 50 *μ*L CaCl_2_ was added. The APTT was then determined.

### 2.9. Cell Culture, Dose-Response Assays, and MTT Assays

MG-63 osteosarcoma cells were seeded at 3000 cells/cm^2^ in 6-well plates under differentiating conditions until the assay endpoint. For dose-response assays, cells were seeded in 96-well plates at 30 000 cells/cm^2^ for 24 h and then dosed with glycosaminoglycans, alternative agents, or controls in 10-fold dilutions from 100 mg/mL to 100 pg/mL. Adenylate kinase activity (for membrane integrity) was measured using the ToxiLight assay kit (Cambrex Corporation) [17].

MTT assay followed the procedures given in the literature [18]. Cell proliferation was measured on A-C, A-NP, and A-CP-F series and on a control glass disc.

### 2.10. Statistical Analysis

Microsoft Excel was used to calculate standard deviations and statistically significant differences between samples using two-tailed Student's *t*-test. For all quantitative assays, each assay condition was performed in triplicate and the results were repeated in at least two independent experiments. A *P* value 0.05 was defined as statistically significant.

## 3. Results

### 3.1. Swelling Ratios Test

**Figure 1:**
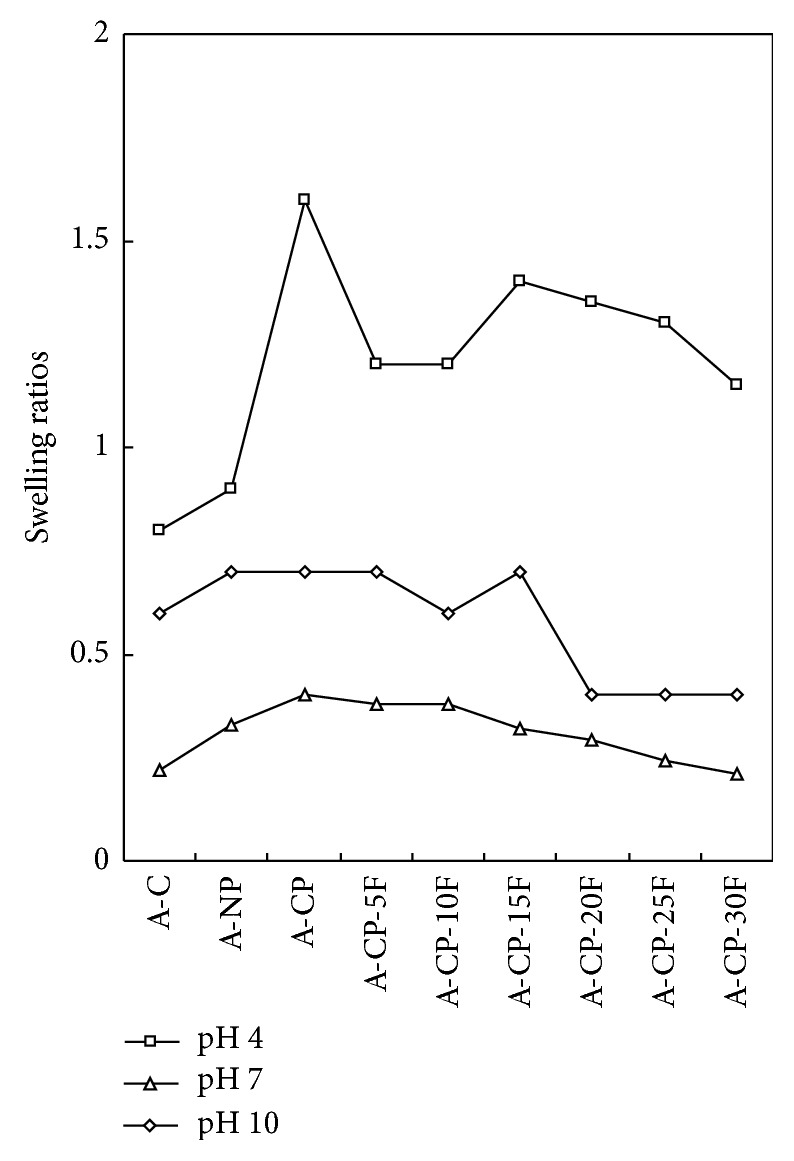
Swelling ratios of A-C, A-NP, A-CP, and A-CP-F series hydrogels with normal saline at different pH.

**Figure 2:**
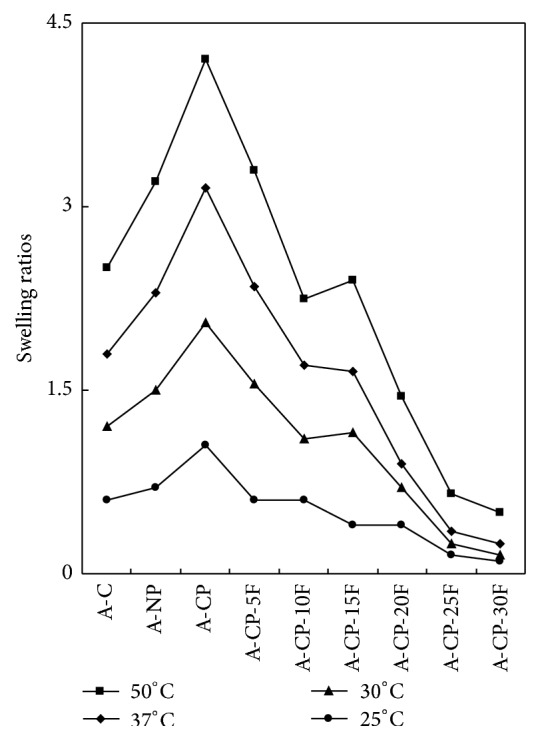
Swelling ratios of A-C, A-NP, A-CP, and A-CP-F series hydrogels in deionized water at different temperature.

The F-127 hydrogel had pH-sensitive swelling behavior. The swelling ratios for A-CP were 1.49 and 0.71 under pH 4 and pH 10, respectively, without F-127, while the average ratios for F-127 hydrogel were 1.21 and 0.52, respectively (Figure 1). The swelling ratio decreased as F-127 increased (Figure 2).

### 3.2. Water Retention Capacity Test

**Figure 3:**
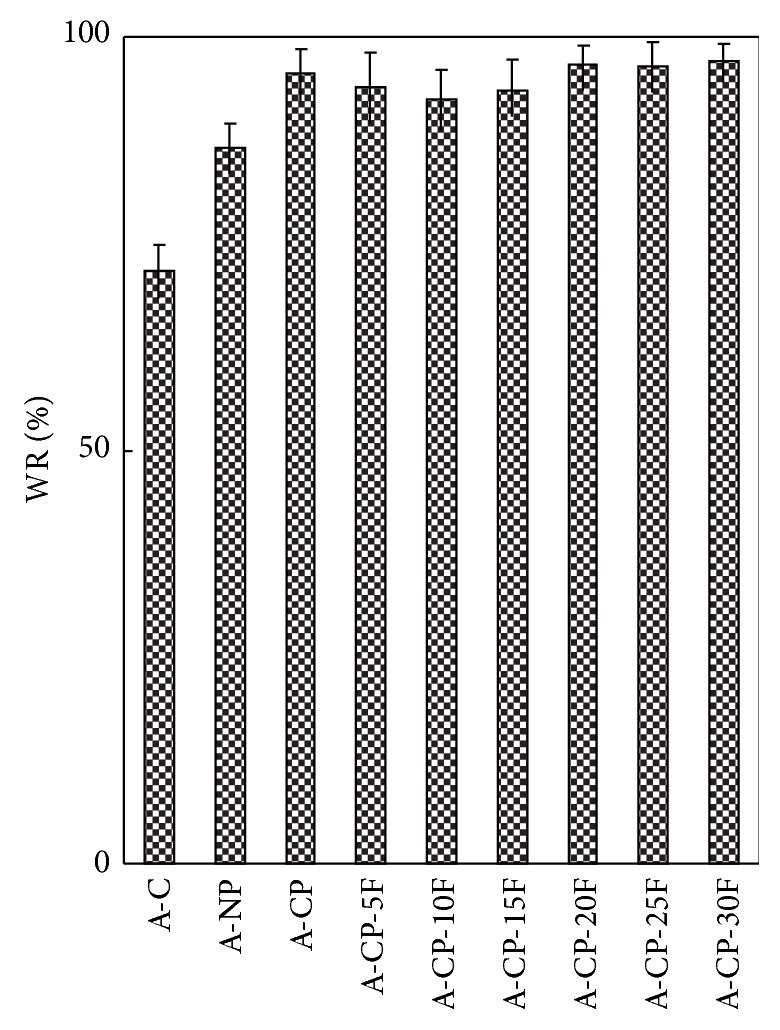
Water retention of A-C, A-NP, A-CP, and A-CP-F series hydrogels with increase in centrifugal force.

The water retention capacity of A-CP was higher than that of A-C and A-NP (Figure 3). This is in the same order as the swelling ratio shown in Figure 1.

### 3.3. Tensile Properties

**Figure 4:**
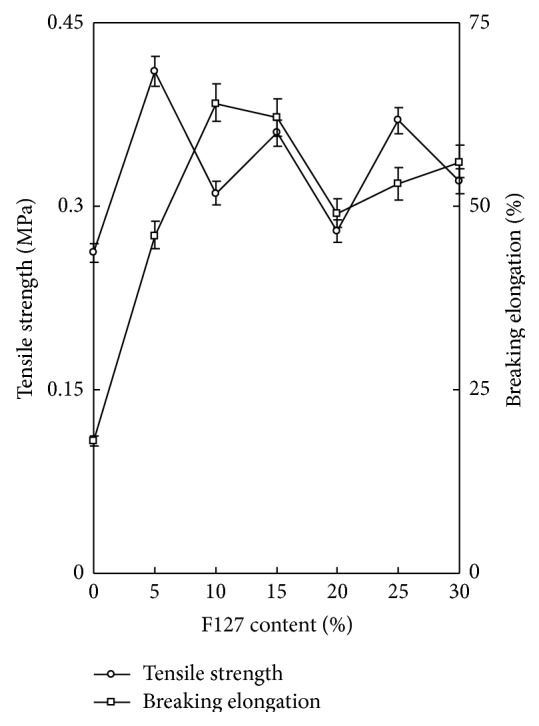
Tensile strength and breaking elongation of A-CP-F series hydrogels.

With progressive 5% increases in F-127 content, the tensile strength was 1.56, 1.18, 1.37, 1.07, 1.41, and 1.22 times that of A-CP hydrogels, while the breaking point was 2.56, 3.56, 3.44, 2.72, 2.94, and 3.11 times that of A-CP hydrogels (Figure 4).

### 3.4. Biocompatibility Test

**Figure 5:**
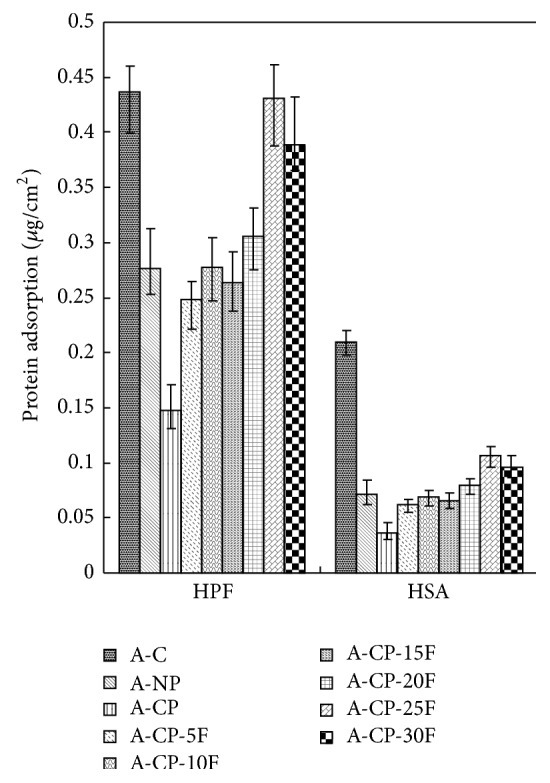
Dependence of adsorption of human serum albumin and human plasma fibrinogen on A-C, A-NP, A-CP, and A-CP-F series hydrogels.

The surface densities of adsorbed HSA and HPF on the A-CP surface were lower than those of A-CP-F and A-C (Figure 5).

**Figure 6:**
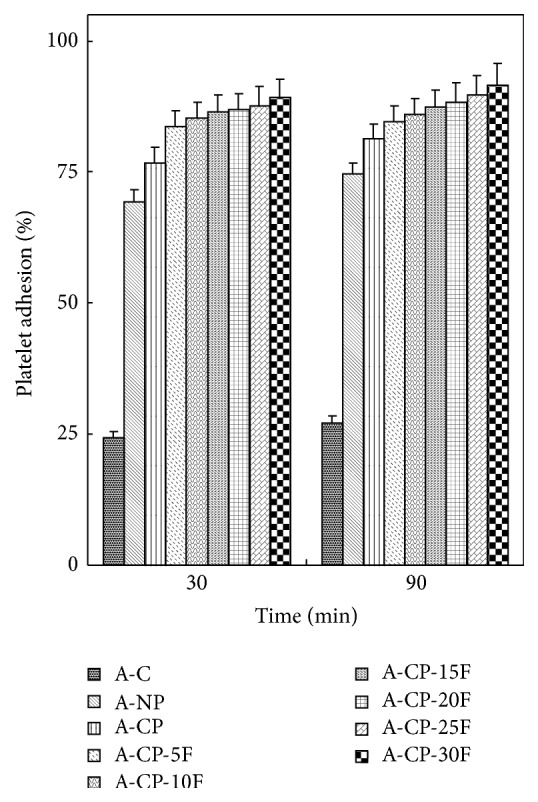
Platelet adhesion of A-C, A-NP, A-CP, and A-CP-F series hydrogels.

After 90 min incubation, platelet adhesion for the A-CP-F series was much higher than that of the A-CP, A-NP, and A-C hydrogels (Figure 6).

**Figure 7:**
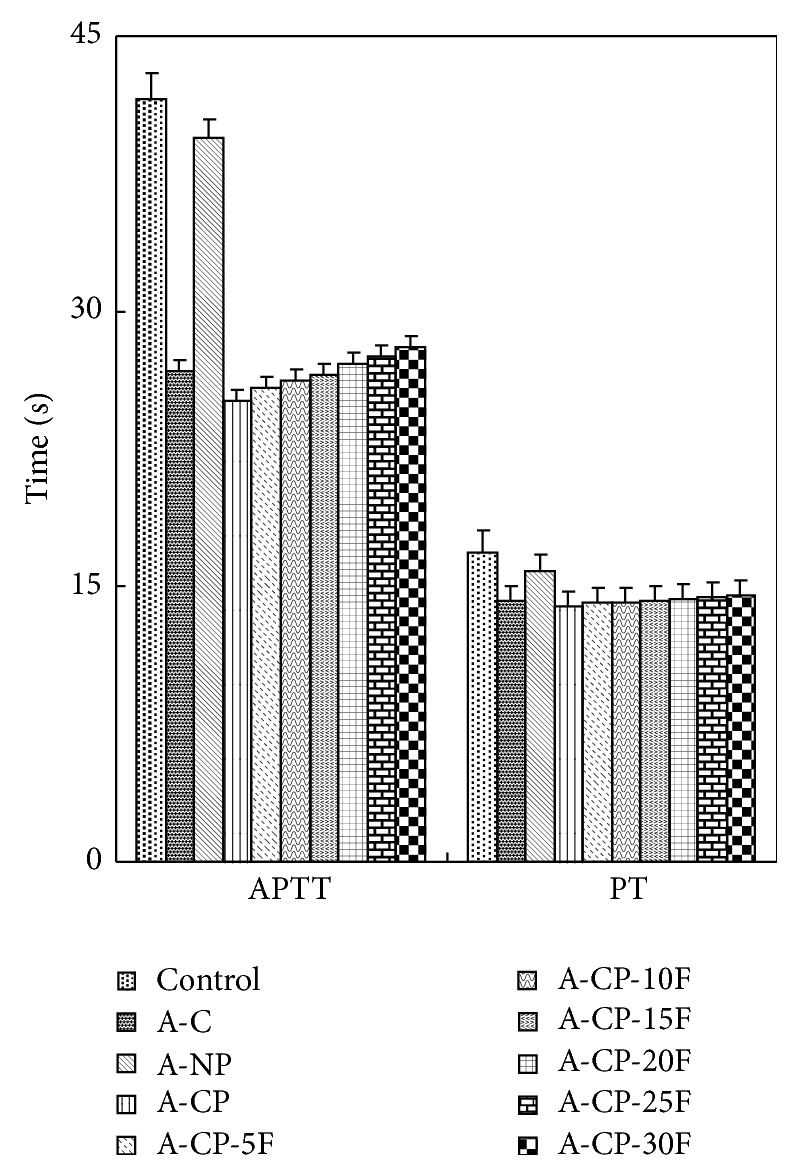
Anticoagulation times of A-C, A-NP, A-CP, and A-CP-F series hydrogels.

The APTT and PT of A-CP-F series hydrogel were about 62% and 84% of that of the blank plasma control (Figure 7).

### 3.5. *In Vitro* Cytocompatibility of the Hydrogels

**Figure 8:**
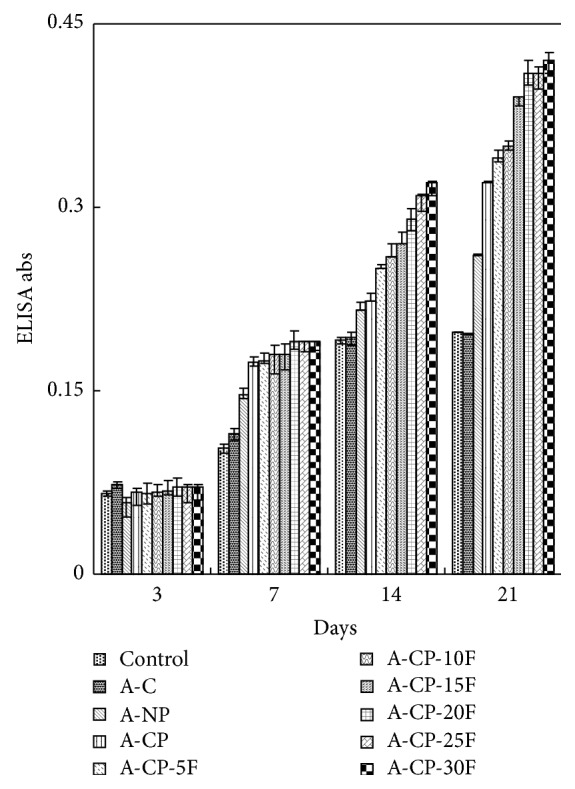
MTT assays of A-C, A-NP, A-CP, and A-CP-F series hydrogels with MG-63 cells.

As shown in Figure 8, MG-63 cells proliferated at a higher growth rate on the surfaces of all the alginate base hydrogels after 3 days. A-NP, A-CP, and A-CP-F series hydrogels all exhibited high viability for MG-63.

## 4. Discussion

Four kinds of alginate hydrogels (A-C, A-NP, A-CP, and A-CP-F series) were compared for mechanical properties, biocompatibility, and cytocompatibility. The swelling ratios decreased as F-127 increased because F-127 strongly interacts with the other compositions and wraps around the polymer chains due to their tight structure. Also, the linear chain of F-127 causes these blends to be more hydrophobic than the A-CP blends [18]. The similarly higher water retention capacities of A-CP and A-CP-F hydrogels suggest that the water retention is related to the hydrophilicity of the hydrogel because of the presence of highly hygroscopic *γ*-PGA. The higher tensile strength and breaking points for F-127 blends compared with A-CP hydrogels indicate that the blending of F-127 caused the surface of the hydrogel to become smoother because of the F-127 polyethylene oxide group [19]. In the adsorption test, the fact that A-CP exhibited less adsorption of serum proteins can be attributed to the carboxyl groups of *γ*-PGA. F-127 has many hydrophobic polypropylene oxide groups. Therefore, the adsorption of HAS and HPF was improved by blending F-127 with A-CP hydrogel [20]. In the platelet adhesion test, adhesion was much higher for the A-CP-F series, which may be attributed to the higher HPF/HSA ratio of the A-CP-F series, which promoted the adhesion of platelets. The APTT and PT of A-CP-F series hydrogels were less than those of the plasma control. Both A-CP and the A-CP-F series hydrogel contain Ca^2+^ (coagulation factor IV), which would activate factors VII, IX, X, and II (prothrombin) [21]. This indicates that F-127 loading does not reduce blood coagulation on the A-CP surface. The test cell lines, MG-63, proliferated on all the alginate hydrogels, A-NP, A-CP, and especially the A-CP-F series, from 7 days of culture, showing that these hydrogels are noncytotoxic and should be a candidate for biomedical applications.

It is known that Ca^2+^ acts as a bridge that triggers the cross-linking and formation of hydrogels, but the higher the Ca^2+^ concentration, the worse the hydrogel. Alginate has hydrophilicity and high swellability originally but only forms a hydrogel with the aid of Ca^2+^, but Ca^2+^ might eliminate useful properties of alginate during the gelation process. Therefore we investigated the exceptional hydrogel formed by mixing *γ*-PGA with alginate; Ca^2+^ still acts as crossing bridge in the preparation process.

Traditionally, alginate gel beads are prepared from sodium or potassium alginate solution in an aqueous solution of calcium ions, typically from calcium chloride (CaCl_2_), to make alginate-calcium chloride hydrogel. The gelation of CaCl_2_ in varying cross-linking densities and a polymer concentration gradient within the gel influences the properties of alginate hydrogels, and the amount and nature of retained liquid substantially affect the porosity and mechanical strength of gel networks as well. Calcium alginate has recently been used as a cell delivery vehicle for* in vivo* tissue engineering research and as a drug carrier that can be easily degraded by enzymes in the organism and eliminated from the body. A major disadvantage to the use of CaCl_2_ is that its gelation kinetics are difficult to control, and the resulting structure is not uniform, so the rate of degradation of typical alginate hydrogels varies. It is improved by joining PGA with sodium alginate solution or calcium alginate. In this method, CaCl_2_ was used as the adjuvant instead.

Hydrogel is used in various ways in medication because, in the swollen condition, its three-dimensional structure is formed by polymer chains with soft and flexible characteristics similar to natural tissue. Good hydrogels have several critical abilities that maintain three-dimensional networks and water retentiveness in use. However, the tighter the three-dimensional networks, the lesser the swellability of hydrogel, but the looser the structure, the poorer the maintenance.

Increasing the calcium content promoted the cross-linking density and reduced the molecular weight between cross-links in the alginate hydrogels. The increased cross-linking density reduces swellability, flexibility, and retentiveness of hydrogels resulting from the more compact calcium linkage network. The copolymerized A-Na-PGA hydrogels and A-Ca-PGA hydrogels overcame the defect because PGA has more hydrogen bonds in the carboxyl group replacing calcium-formed ionic networks. The carboxyl groups on the polymer chain residues of PGA are highly sensitive to pH, which creates flexible, highly absorbing, and smoother hydrogels. All the investigations of alginate hydrogels suggest that these hydrogels, especially A-Ca-PGA hydrogels, can lead to successful application for medical and pharmaceutical utilization.

## 5. Conclusion

As calcium plays a major role in bone metabolism, Ca-PGA has a positive effect on the reconstruction of bone. Therefore these A-CP-F series gels are applicable as injectable bone repair material. The molecular weight between cross-links and the cross-linking density of the hydrogels were characterized from the equilibrium swelling theory. Increasing the calcium content increased the cross-linking density and reduced the molecular weight between cross-links in the alginate hydrogels. Therefore, the A-CP-F composite could serve as a useful bone substitute for repairing bone defects.

## Figures and Tables

**Table 1:** Preparation and composition of hydrogel blends.

Ingredient	Designation
Alginate	
CaCl_2_	A-C
Na-PGA (with CaCl_2_)	A-NP
Ca-PGA	
0% F-127	A-CP
5% F-127	A-CP-5F
10% F-127	A-CP-10F
15% F-127	A-CP-15F
20% F-127	A-CP-20F
25% F-127	A-CP-25F
30% F-127	A-CP-30F
